# Report on Operative Dentistry

**Published:** 1871-02

**Authors:** Geo. H. Cushing


### ARTICLE II.

### *Report on Operative Dentistry.*

Read before the American Dental Association.

By Geo. H. Cushing, D. D. S.

A report upon operative dentistry should naturally present all that has been done in this department since the last full report was made, whether in the matter of improvements, or changes both as to methods of practice and materials used and instruments and appliances introduced, tending either to simplify operations or to render them more nearly perfect.

The endeavor will be made in this report to do this as briefly as is consistent with the importance of the points to be considered. But it must not be inferred that the report lays claim to perfection, either in regard to the introduction of every modification in practice or to the manner of treatment of the subjects considered.

While it is possible that it may treat of some things which are unimportant, it is very probable that some things will not appear that should have a place in it, from the want of information or forgetfulness of the committee.

The subject of operative dentistry is one of such importance that reports upon it might naturally be expected to be found occupying a prominent place in the transactions of this association, but a search of the records would certainly not convey to the investigator the idea that it was of grave importance, as only two reports upon it are to be found since the organization of the association, and neither of these could in any sense be said to fully cover the ground which such a report should cover.

It has been deemed proper to make this report, if possible, a compendium of the main features which have marked the progress of operative dentistry since the formation of this association, and for the reason, that while much that has been done and many of the improvements made during that time have been fully discussed and written about in our various societies and jonrnals, yet there is no report as such among the transactions of the association covering all the ground. The propriety of having such a record among our transactions must plead the excuse, if such is needed, for referring to points generally understood, or which have been previously written upon.

It would seem proper, for the purpose of securing the greater completeness of this report, to speak first of the use of cohesive foil for the purposes of operative dentistry, because of its being the first snbject in the order of time, and because since the time of its introduction it may be claimed that the most marked advances in operative dentistry (as applied to the preservation and filling of teeth) have been made which the profession has ever known.

Since the introduction of cohesive foil to the attention of the profession by Dr. Arthur, some fifteen years since, it has steadily been growing in favor with practitioners generally; and it may now be said that the method of filling teeth with cohesive foil has been generally adopted.

That this method is more in accordance with philosophical principles than any other, would seem to be a fact easily to be established. In the use of soft foil exclusively, the principle of wedging must be depended upon entirely for the security of the filling, and in order to make a dense and thorough operation, great wedging force must necessarily be used. Great judgement is requiste to determine the amount of such force a tooth will safely bear, while in some cases the frailty of the walls is so great that any amount of such force necessary to properly secure a filling would seem likely to prove fatal to the integrity of the tooth. The least amount of such force requisite to properly pack, secure and thoroughly condense a filling that can be applied to a tooth must be acknowledged to tend to some extent to its disintegration and to fracture; and inasmuch as more force is generally used than is absolutely necessary, the certainty of weakening the tooth and the danger of accident from fracture render the objections to the wedging process quite serious. All practitioners probably have seen numerous instances in support of this position where teeth had been fractured and injured by this wedging force.

In the process of filling teeth with cohesive foil, the principle involved is entirely different. The filling should depend mainly upon secure anchorages, made by means of retaining pits, formed at the base of the cavity and elsewhere where the dentine is firm and strong. From this base or anchorage the structure of the filling proceeds with almost the entire force necessary to the condensing of the gold, directed in the line of the longitudinal axis of the tooth and root, the lateral force necessary to be used as compared with the wedging force when that method is followed, being very slight indeed. This method must clearly occasion less disturbance of the structure of the tooth, less liability to accident from fracture during the operation, and less weakening of the tooth from lateral pressure upon the walls, while the filling may be more thoroughly secured and more densely compacted than by the wedging process. It would seem clearly true that this is the more scientific of the two methods, where either might be used. But there are cases in which the wedging process could not be used, as where it is impossible to so shape a cavity that it shall present two or more opposing walls nearly or quite parallel to each other or where the walls are so thin as to render sufficient wedging force impossible or highly dangerous, or in building out beyond the margin of the cavity, In such cases the use of cohesive foil enables the fulfiillment of the requirements of the case as nothing else could do. It renders possible the performance of operations which otherwise could never have been performed, and secures the preservation of a large class of teeth, which otherwise would of necessity fall prematurely a prey to the forceps.

The introduction of cohesive foil, then must be regarded as a decided improvement, and the influence which this innovation has exercised in developing all the improvements in practice which have marked the period since it occurred can hardly be realized. It is not intended by these remarks to ignore the various forms of sponge or crystal gold. They will be referred to in their proper place. But the report proceeds upon the basis that cohesive foil better fulfills all the conditions demanded generally of a material for filling teeth than any preparation of gold.

A marked advance is clearly perceptible in the last few years in the matter of thoroughness in the preparation of cavities.

The more careful preparation of the margins of cavities and the more complete removal of affected and imperfect tooth substance, whether absolutely broken down or not, has evidently received far more attention than formerly. The necessity for cutting out fissures and making complicate cavities where there are several simple ones in the same tooth, with but slight sound structure intervening, is growing daily more evident to the profession, and the advanced benefits arising from this advanced mode of practice are becoming more and more established in the minds of all careful observers.

The great principle of physiology and pathology, an inteligent application of which is so essential to successful operations in the mouth as well as elsewhere, are generally better understood, and influence the intelligent practitioner of to-day in all his operations to a greater or more satisfactory extent than ever before, adding to his success in just the ratio in which his familiar acquaintance with these principles approaches thorough understanding of the ultimate truth.

These causes above noted combined have tended to give an impetus to conservative dentistry in the broadest sense of that term, which is truly gratifying and gives the highest promise for the futnre.

Under this impetus renewed efforts have been made to develope a safe and satisfactory practice for the preservation of the life of the pulp in cases where it has become nearly or quite exposed. In this report it is only proper to speak of two methods looking towards this end which have received attention of late years, viz: the method known as "amputation of the pulp," and that of "capping with oxy. chl. zinc." The first named of these methods claimed the renewed attention of the profession some four or five years ago, by the improved operation as devised by Dr. W. W. Allport, of Chicago. This improved operation consisted in excising a portion of the pulp and in trimming and smooothing the edges of the orifice of exposure, thoroughly removing the debris, so that no irritation could arise from roughened edges, or from spiculæ of bone impinging upon the delicate structure of the pulp. The careful capping of this after the operation with bibulous paper, and filling over that with Hill's stopping, left the parts to heal and to throw out a deposit of secondary dentine for their own protection.

This operation is doubtless in harmony with physiological and pathological laws, and under the hands of its originator was unquestionably successful to a certain extent, but the very great delicacy of manipulation necessary to secure good results, and the limited number of cases of exposure in which it could be at all applied would seem to render it of no practical value to the profession generally.

The method of capping with oxy. chl. zinc appears to have been in use with Dr. Keep of Boston as long ago as 1855, and so far as is known he was the first to use oxy. ch. for this purpose, though others may have also experimented with it as early as that who are not known to the committee.

Dr. Keep called the attention of the profession to this method in a paper sent to the dental convention which met at Saratoga or Niagara in 1858 or 1859, entitled "A new and successful method of filling teeth with gold over exposed nerves. By N. C. Keep & Son.

For some reason but little attention seems to have been paid to this method by the profession generally until some time after the meeting of this association in Boston in 1866, when Dr. Atkinson, of New York, called attention to it through the dental journals, claiming remarkable success for it. The method generally in use probably is in the main that suggested by Dr. Atkinson, viz: of thoroughly removing all diseased dentine from over the pulp, even to causing the pulp to bleed, applying creosote, chloroform, aconite, or morphine to subdue the pain; after drying thoroughly the cavity, applying a small drop of creosote immediately to the exposed point, and then flowing the oxy. chl. over that; after the thorough settling or hardening of the oxy. chl., the cavity either to be permanently filled with gold or temporarily with Hill's stopping. This method of treatment with oxy. chl. zinc has probably been more generally tried than any other ever before introduced, and with a degree of seeming success that warrants the hope that with such modifications of practice as may come in the future as the result of study and experiment in this direction, it will be found that the great desideratnm has been attained of a practice whereby the vitality of the pulp of the tooth may be preserved after absolute exposure even of long standing. Yet until the nature of the action of this remedy is better understood, and until a longer time shall have furnished its evidence regarding this practice, success can not be absolutely affirmed.

There are some of our best men who claim for it complete success with scarcely any exceptions. Others again, equally reliable and careful as practitioners, assert that the death of the pulp ensues sooner or later in almost all cases. But the weight of the testimony seems to be in favor of its success. While this is so, it would be clearly unwise to reject the practice because of some unfavorable results, until a longer time shall have elapsed as the test of those earlier operations which up to the present moment certainly have proved successful. The mind of the profession should rather be bent upon the study of the subject in order that the remedy and its action may becomo thoroughly understood, so that those modifications of practice may be determined which shall give greater certainty to the practice in the future.

It will not be attempted in this report to explain the action of this remedy, but only briefly to refer to certain observed facts concerning the practice, and to make some general suggestions concerning the details of the operation and the cases in which most hope of success may be indulged.

That a deposit of secondary dentine or calcific material does take place in some cases after treatment by this method there is ample proof, numerous instances being vouched for by some of the most reliable men in the profession, but how generally this takes place of course can not be known, as comparatively very few cases are examined with reference to ascertaining this point. It has also been demonstrated that many teeth having been thus treated have evinced satisfactory evidence of the vitality of the pulp after a lapse of two or three years or more, upon the application of ice in direct contact with such teeth.

Again, it has been affirmed by a class of observers in certain localities that in most cases in which the pulp has been found to be dead after this treatment, there had been little or no pain or soreness or disturbance of any kind after the operation, and that when the pulp chambers of such teeth were opened, they and the root canals were found to be entirely empty and free from moisture or evidence of decom. position. These seem to be the prominent and noticeable facts concerning this practice, and in view of the last mentioned might it not be suggested in answer to the objections urged by some "that the pulp is certain to die sooner or later in most cases;" that if it dies in the manner as claimed by some as above stated, and the interior of the tooth is found in the condition named—is it not then in better con dition than it would likely to be in, in a majority of cases, had the pulps been destroyed in the usual manner?

With reference to the details of the operation, it should be strenuously insisted that the greatest thoroughness is essential in removing all diseased structure and cleansing the cavity of all debris, that no irritation may arise from those causes after the completion of the operation. Again, great care should be exercised to avoid irritating the pulp beyond what is absolutely necessary, and in applying the oxy. chl. zinc it should be allowed to flow over the exposed point, and should never be pressed until it has thoroughly hardened. To the neglect of some one of these vital points may unquestionably be traced some if not most of the fail ures which have occurred in cases of recent exposure.

The great irritation and pain produced by the immediate contact of the freshly prepared oxy. chl. with the exposed pulp, even when a layer of bibulous paper saturated with creosote is interposed, would suggest the propriety of the adoption of some mode which should lessen, if possible, this pain and irritation.

In this connection the practice of Dr. Geo. O. Howard, of Galena, Ill., is worthy of trial. He uses a preparation of which the following is the formula:

Saturated alcoholic solution of morphia, one part; three and one half parts ether. In this mixture dissolve as much gun cotton as it will take up. With a camel's hair pencil he applied a few coats of this over the point of exposure before flowing over the oxy. chl. zinc.

This preparation he claims adheres closely to the tooth and pulp, forming a pellicle that will not crack, and under which in some cases, with the aid of a lens, the pulp may be observed distinctly to throb.

This he claims prevents to a great extent the pain which generally follows the application of the oxy. chl. zinc. The cases in which treatment by oxy. chl. is most successful are unquestionably those of quite recent exposure, where there has been little or no pain previous to the operation. The cases likely to prove less successful are those in which the pulp has been long exposed, with more or less pain, but with slight disorganization of the structure of the pulp; and those cases least likely to succeed are where the pulp is more or less ulcerated or gangrenous. In the latter cases perhaps the best practice is to secure, if possible, by treatment with creosote, or otherwise a sloughing off of tne diseased portion of the pulp; when, after that has taken place, careful manipulation and thoroughness in details, as in the cases of recently exposed pulps, may give some hope of success.

In concluding these remarks upon this subject, it should be observed that the operation under discussion is one requiring great nicety of manipulation and an understanding of what is being done, and it would be absurd to expect uniform results in miscellaneous practice, when so great a "diversity of gifts" must necessarily exist among the profession at large.

But the conclusion must be forced upon every candid mind that in view of the general testimony in its favor, the practice of capping exposed pulps with oxy. chl. zinc has been proved to be the most successful thus far introduced to the profession tending to the preservation of the life of the dental pulp, and may justly claim to be a very important adjunct in operative dentistry. It certainly demands the careful, thorough study and experimental investigation of the profession as giving promise of the highest results in the direction of conservative practice.

We come now to the consideration of the somewhat startling views of Dr. Arthur, as promulgated in his book published a few years since, in which he advocates a very marked modification of general practice for the preservation of teeth, and insists upon a heroic use of the file as the surest means of preventing and arresting decay in these organs.

Startling as these propositions doubtless were to the profession generally, the careful consideration of them may show them to be not entirely devoid of reason. In advocating such a practice, the author has to contend against the strongest prejudice probably which ever settled itself in the minds of men, both with certain members of the profession who are numerous, and with a large portion of the public.

That Dr. Arthur's views are extreme there can be no doubt. No man would be likely, after elaborating in his own mind such a system, as the result of years of study and observation in that particular direction, to announce it to the profession without occupying very extreme ground ; but that all his propositions should be characterized as wild and unworthy of thoughtful consideration, does not follow at all as a matter of course. This report could hardly claim to aim at completeness did it ignore Dr. Arthur's views, as although they can not be said to be fully adopted by the profession, or the practice they advocate to have become to any extent general, yet there can be no doubt but that practice generally will be largely modified in the coming years through their influence.

Dr. Arthur presents for our consideration a system, the result of long and careful observation and experiment, and sustains his views by much sound reasoning and some very plausible arguments. Views presented under such conditions are always entitled to thoughtful consideration, however erroneous they may appear at the first glance, and it would be strange indeed if no good were to be found in them, if the search instituted be after truth.

That the teeth may be filed away to a considerable extent without greatly increasing their liability to decay, it is believed admits of no question. The proposition that dentine, if sound and thoroughly polished, will resist destructive agents nearly if not quite as well as enamel, can hardly be controverted, and that judicious filing will not only arrest but prevent decay to a great extent, must be apparent to every observing practitioner of long experience. That all the teeth back of the cuspids should be separated with the file, whether decayed or not, in case the incisors are decayed before the twelfth year, this report is not prepared to admit, but that a more general and more thorough use of the file as a means of prevention, would, under Dr. Arthur's method, in certain cases, prove of vast benefit, there can be no doubt. Accumulated experience seems to tend to confirm this view, and it is not improbable that the next ten years will demonstrate to a large extent the soundness of the major part of Dr. Arthur's system.

It must be understood that this report does not admit the propriety of *indiscriminate* filing, but of *judicious* filing, and it should be remembered that certain classes of teeth under certain conditions will scarcely admit of filing at all; certain others of filing only to a limited extent, while some may be filed away to a very great extent without injury or serious objection. It should ever be borne in mind that in speaking of operations or methods, reference is made to their performance as they should be performed, thoroughly and perfectly, and in considering the different modes of practice we should not lose sight of the fact that because certain modes fail in some hands they do not necessarily fail in all. It is not unreasonable to suppose that where failures are the rule with some, and yet that the same operations are generally successful with others, that the failures are largely due to the absence of some of the conditions within the power of the informed and skillful operator to supply, which are essential to success.

In coming to the consideration of the latest and most marked change in practice in regard to filling teeth, that is the substitution of heavy foils for those formerly in use, we are brought to the discussion of the most complete revolution in ideas and practice that the profession has ever witnessed. So great was this revolution, that although the fact is established beyond controversion that heavy foil is the material above all others for securing the best resnlts, yet its advocates have hardly ceased to wonder that so great a change could have become so quickly established or to so general an extent as this unquestionably has. When Dr. Atkinson suggested the use of heavy foil one year ago, there were probably very few in the profession but that regarded the proposition as the vagary of an enthusiast. To-day the practice may be said to be becoming very general.

When it is borne in mind that the tendency for some years has been towards lighter foils, and that many of our best operators regarded No. 2 as the best adapted to secure the highest results and when we consider that this change in practice proposes to substitute for numbers 2 to 6, numbers 30 to 240, the magnitude of the revolution becomes apparent. But in spite of the seeming absurdity of this proposition, it is believed that there are sound philosophical reasons to support the claims made in behalf of the heavy foils.

The endeavor will be made briefly to state these reasons and to further state what experience seems to have established concerning their use. To reconcile this proposition with the new principles of philosophy, it is only necessary to consider how light foil is used. It is prepared for filling by being folded upon itself a greater or less number of times, according to the mass required and the particular use to which it is to be applied. This is the manner of its preparation whether used in pellets, blocks, cylinders or strips. It present then, when thus folded a mass from ten to forty times as thick as the foil from which it is prepared. If prepared from No. 4 foil it would represent by multiplication of thickness Nos. 40 to 160 of heavy foil. If then, ten to forty layers of No. 4 foil can be packed successfully, why not one layer of 40 to 160. Practically, this would be more easily accomplished, because when light cohesive foil is thus folded upon itself, it is always with a greater or less degree of irregularity, and the force required to condense a mass thus prepared must necessarily be greater than for a single layer of same thickness, because the mass of folded foil presents innumerable surfaces and angles braced in every direction against each other and cohering at every point of contact, which offer a much greater resistance than a single layer corresponding in thickness would do, while there are largely increased surfaces to weld together.

It would seem to be unnecessary to say another word in support of the philosophy upon which the claims for heavy foil are based.

With regard to what experience has demonstrated concerning their use, it may be said that as high as No. 60 is applicable to all cases where the mallet can be used, even in the most frail teeth, when those experienced in its use would think it must surely fail. But it seems to be establisned that foil as heavy as No. 60 is peculiarly adapted for use against frail walls, that it is in fact vastly superior to the light foils. It accomodates itself to irregularities with great facility, requiring but slight force to place it properly and thoroughly in position, and in fact gives immediate support to the frailest wall, and renders operations in such cases less difficult and dangerous. No. 60 for filling retaining pits is much better than lighter numbers, making more certain and solid anchorages.

The higher numbers of foil are very much more cohesive, and consequently when properly packed must make a stronger as well as a more solid filling, while owing to this greater cohesiveness as well as the greater softness of numbers as high as 60 and upwards, really less force is required to condense them thoroughly than the numbers from 2 to 20.

In one class of cases the heavy foil seems to possess peculiar value in binding one part of the filling to another. For instance, where a cavity in a bicuspid or molar extends from the approximal surface over to the crown, where the approximal cavity may be very shallow, and as is frequently the case, it is impossible to secure thorough anchorages either at the base of the cavity or elsewhere, but where strong anchorage can be obtained in the crown cavity. In such cases, after securing as thoroughly as possible the base of the filling in the approximal cavity, and thorough anchorage in the crown, a narrow strip of No. 120, well secured to the crown filling and carried over to the approximal portion, and so carried back and forth two or three times, will secure the approximal portion as no other method could possibly do. So, too, in building across the ends of the incisors where there are approximal cavities running into the grove across the cutting edges or surface; this method secures the approximal fillings from any possibility of becoming displaced. In this class of cases it is believed that the heavy foils enable the performance of operations of a more reliable and durable character than could be attained with any other material. In building out and restoring the contour of the tooth, especially on the cusps and across the cutting edges, where great strain comes upon the filling, the much greater strength which the heavy foil gives, is invaluable, and renders such operations less experimental than they have some times been considered when made with the light foil.

One feature peculiar to heavy foil deserves especial notice, and that is that it spreads more laterally in coudensing than the light, a fact which should be carefully borne in mind, as the same mallet force applied throughout an operation with heavy foil, as would be used with the light, would in some cases fracture the tooth, and often check it to a greater or less extent. To sum up in a word the merits of heavy foil, it makes when properly used the most perfect operations that have yet been attained.

It is objected by many that the profession is wild about this matter, that too much is claimed for this practice, and that until the test of time has stamped it with the seal of approval, it should be claimed as a success.

This would seem to be a not unreasonable proposition, and as to new methods and practices generally is unquestionably a proper caution to observe; but the matter under consideration seems to be exceptional in that it carries conviction of its permanency with it. It seems to be susceptible of demonstration to those who have used the heavy foils that there can be no failures chargeable to the material or method—that the assurance of permanency is as positive now as though years had added their testimony.

It can not be feared for this material, as in the case of "plastic goid, that after a time fillings would be found to be disintegrating and crumbling away and failing at the mar gins." Gold plate does not disintegrate, and if heavy foil is properly placed over the margins of cavities, there can be no crumbling at that point. These operations have been submitted to the severest tests possible, except that of time, and the móst powerful lenses and the most delicate points fail to discover any evidences of weakness or failure, while the working of the heavy gold carries conviction to the mind of the operator, as the working of no other ever did, that strength and durability are the marked characteristics of the new method of practice. It has been urged by some that they fear by and by it will be found that fillings are falling out, or leaking at the margins. Have the gentlemen who have these fears never seen failures or leaking at the margin from the use of light foils? There has not yet been discovered any material for filling teeth that renders unnecessary the use of brains.

It is not expected that the highest results will be reached with heavy foils through careless or slipshod operations. But it is believed that thoroughness and skill will produce higher results with heavy than with light foil; that those who produced fillings with light foil that did not leak at the margin, will produce them with more certainty by the use of heavy foil; and those whose fillings of light foil did not drop out will make them of heavy foil with greater promise of durability.

Perhaps not the smallest merit of the heavy foils is that of the almost impossibility óf the conscientious operator making an imperfect operation without knowing that it is so. Very many operations conscientiously performed with the light foils have failed because of the deceptive appearance and imperfect evidence of thoroughness to the operator in the various stages of the operation. In condensing masses of light foil it is very easy to be misled by superficial appearances, as a pellet of light foil may sometime seem to be thoroughly condensed and to have cohered throughout its whole mass, which is only superficially condensed and is attached to the filling by few and largely separated points of cohesion, while such is hardly possible to occur with the heavy foil.

Every step of the operation must be thoroughly performed, and the operator is aware of this fact from the evidence continually before him.

(*To be continued.*)

---

